# Biological approaches for addressing the grand challenge of providing access to clean drinking water

**DOI:** 10.1186/1754-1611-5-2

**Published:** 2011-03-31

**Authors:** Mark R Riley, Charles P Gerba, Menachem Elimelech

**Affiliations:** 1 The University of Arizona, Tucson, AZ, 85721, USA; 2 Yale University, New Haven, CT 06520-8286, USA

## Abstract

The U.S. National Academy of Engineering (NAE) recently published a document presenting "Grand Challenges for Engineering". This list was proposed by leading engineers and scientists from around the world at the request of the U.S. National Science Foundation (NSF). Fourteen topics were selected for these grand challenges, and at least seven can be addressed using the tools and methods of biological engineering. Here we describe how biological engineers can address the challenge of providing access to clean drinking water. This issue must be addressed in part by removing or inactivating microbial and chemical contaminants in order to properly deliver water safe for human consumption. Despite many advances in technologies this challenge is expanding due to increased pressure on fresh water supplies and to new opportunities for growth of potentially pathogenic organisms.

## Introduction

Water scarcity is a fact of life in arid and semi-arid regions where agricultural, domestic and industrial demands compete for limited resources. Access to clean drinking water presents a monumental challenge that is well documented for the developing world but is a rising problem for more established regions [1]. The problems for both locations are often presented in simplified form as being either a lack of water quantity or a lack of water quality; however, the reality is infrequently so straight-forward. The NAE Grand Challenge document http://www.engineeringchallenges.org/cms/8996/9142.aspx states:

"Lack of clean water is responsible for more deaths in the world than war. About 1 out of every 6 people living today do not have adequate access to water, and more than double that number lack basic sanitation, for which water is needed. In some countries, half the population does not have access to safe drinking water, and hence, is afflicted with poor health. By some estimates, each day nearly 5,000 children worldwide die from diarrhea-related diseases, a toll that would drop dramatically if sufficient water for sanitation was available."

In the so-called developed world, an aging infrastructure plays a large role in problems of providing clean drinking water. Along much of the U.S. east coast, conveyance systems (pipes, pumps, valves, etc.) were designed for a 100-year life span, but were constructed in the middle 1800's. Much of the water infrastructure is of poor quality, which has led to substantial leakage of water and unaccounted-for water totaling one half of that initially introduced. In many cases drinking water pipes are located in close proximity to black water (wastewater) pipes. In a highly connected piping network, pressure can at times be either positive or negative relative to the surrounding water table. In a leaky system, drinking water can mix with a variety of water sources, thus providing one common route for introduction of microbial contaminants into the drinking water supply.

Certainly, the world does possess sufficient water for all of man's endeavors. Globally, water is available in abundance. Unfortunately, water abundance does not match locations and the quality to meet mankind's need. Even within close geographic locations, some regions may be awash in fresh water while other regions, perhaps separated by only a few hundred miles, are afflicted by drought. In many instances, political and economic barriers prevent access to water even in areas where it is otherwise available. "Overcoming the crisis in water and sanitation is one of the greatest human development challenges of the early 21st century," a recent U.N. report warns [2].

Public perception of drinking water and its value is complex. Nearly half of the U.S. population avoids drinking tap water [3] and only 40% of respondents to a survey are reportedly willing to contribute some amount of money for improvement of drinking water quality [3]. The majority of these refusals are characterized as protest responses. Surprisingly, the frequency with which an individual drinks tap water does not influence their willingness to support drinking water quality improvements. There are reports [4] that a substantial proportion of the deficiencies in drinking water systems contributing to consumer complaints and waterborne-disease outbreaks are associated not with the conveyance systems, but rather are due to problems in the premise plumbing (that is, plumbing within the home or industrial facility).

Water for drinking and personal use is only a small part of society's total water needs with household water accounting for generally less than 5 percent of total water use. Most water is utilized for agriculture and industry, not accounting for water needed for natural ecological processes. The oceans contain an enormous reserve of water which could be made potable through desalination processes such as those in use in many arid regions, but cost currently limits their use in areas of moderate need. Substantial progress has been made in the past 10 years in improving desalination methods and developing the technology to function on a large scale [5]; however, improvements are still needed and uses for the brackish effluent must be further developed so as not to contaminate naturally available drinking water supplies. In some developing countries, water supplies are contaminated by human wastes (fecal matter and endocrine disrupting compounds), industrial and agricultural wastes (pesticides, solvents), naturally occurring contaminants (arsenic, lead, and other metals), and increasingly due to the results of natural disasters and industrial accidents (typhoons in Southeast Asia; oil drilling in the Gulf of Mexico).

This report will focus on challenges and a few biologically-based solutions to address water quality and water quantity. Specifically we shall address microbial contamination as a water quality problem and the use of desalination as a means to address water quantity limitations.

## Waterborne pathogens

Meeting the need of providing adequate drinking water has separate tasks based on location and degree of living conditions. The developing world has high childhood mortality with high endemic disease and a need for low cost sustainable treatment [6,7]. In the developed world, outbreaks and special populations create endemic and chronic diseases. Every time we change our environment, we create new opportunities for new types of organisms to thrive. When we create a new source of water for human use, we open the environment to new types of organisms that might have negative effects on human health and on the natural environment. There is a need for real time monitoring and management of water treatment and water distribution systems that are currently monitored through infrequent batch measurement procedures, which unfortunately, can miss transient, but problematic water quality events. The EPA assessed how much illness from tap water occurs in the U.S. from multiple studies conducted over a similar time frame. Studies reported that from municipal water systems alone there were between 12 - 19.5 million cases of diarrhea/yr [8-10]. In 2009 Gerba estimated that there were 7,000-20,000 deaths/yr from water-based organisms (unpublished). To place this in context, there were more people who died from water-based diseases then died from AIDS in 2008.

Recent studies on over 500 wells around the U.S. showed that 10-42% of groundwater supply wells are contaminated with enteric viruses [10]; 95% of all individual and small groundwater systems violate one or more primary and secondary drinking water standards in Arizona [11]; and 52% of all point-of-use carbon filters contain an enteric organism after 3 months of use in a municipal system [12]. Approximately 80% of consumer complaints about tap water quality originate in the consumer's home distribution system [4], indicating that management of water quality problems cannot easily be addressed from a single central location.

A broad range of treatment options are available for decentralized sewage systems, but high rates of failure of traditional septic tanks [13] discourages innovation in favor of centralized sewerage installation. Oftentimes, indicator organisms or surrogates are applied for monitoring water quality and developing water treatment methodologies; however, the scientific community does not have consensus on how well these indicators truly reflect the behavior of target organisms, which may be pathogenic, teratogenic, endocrine-disrupting, or problematic in other ways.

Even though potable water distribution systems present a hostile environment for growth of microorganisms due to low nutrient levels as well as the presence of disinfection residuals [14], biofilms form ubiquitously and may provide a near constant source of HPC (heterotrophic plate count) bacteria even when the water has been subjected to disinfection processes including chlorination, chloramination and ultra-violet (UV)-treatment [15,16]. Heterotrophic bacteria are those that require organic carbon rather than carbon dioxide as a carbon source; all human pathogenic bacteria are heterotrophic. While disinfection residuals within distribution systems reduce the potential for growth of pathogens and regrowth of heterotrophic bacteria, they do not eliminate them [17]. While the presence of heterotrophs does not in itself constitute a human health hazard, they can serve as a means to assess the microbiological quality of the water and the efficacy of water treatment [18].

The U.S. Environmental Protection Agency (U.S. EPA) has recommended that heterotrophic bacterial numbers in drinking water should not exceed 500 colony-forming units (CFU/mL), primarily because of the interference of coliform detection [19]. This number is based on the effect of HPC populations on recovery of total coliforms rather than on a health-based action level [20]. Higher numbers of HPC are often the result of bacterial regrowth, particularly in the distribution system [21] and in the water treatment devices mounted at the household tap [22]. It should be noted that in an evaluation of the relative contribution of heterotrophic bacteria from various sources in the normal diet of an average person in the United States, less than 5% of the average consumer's total heterotrophic bacteria intake was found to be derived from drinking water [23]. However, individuals in high-risk categories, such as the immunocompromised, young children, or the elderly could be at risk from the consumption of drinking water containing opportunistic pathogens that are members of the HPC group [24].

According to Allen and coworkers [20], the number of HPC bacteria in drinking water varies widely, depending on many factors. These include the initial quality of the source water, the types of treatment, and the type and concentration of disinfection residuals. Additionally, water age and ambient temperature of the raw and finished water influence HPC levels.

Based on these points, HPC are often used as an indicator to assess the microbial quality of drinking water even though the clinical and epidemiological evidence that HPC bacteria pose a health risk is lacking.

## Methods to address waterborne pathogens

A number of methods are in development for removing or inactivating waterborne pathogens. Here we focus on a few instructive examples.

In 1993 the largest waterborne outbreak of disease ever documented occurred in Milwaukee, Wisconsin. Over 400,000 individuals developed gastroenteritis and perhaps 100 individuals died in response to consuming drinking water contaminated with the protozoan parasite *Cryptosporidium parvum *[25]. The organism appeared in high concentrations in the city's water source after a period of heavy rains, allowing some of the oocysts of the organism to penetrate the filtration barrier. Infections ranged from acute conditions, such as diarrhea, to chronic conditions with symptoms that remained present even 10 years after initial infection. Chlorine disinfection had little effect on the viability of the oocysts, and in fact, this organism was later detected in 60% of the treated drinking water supplies in the United States. Since that time, new rules for protecting surface water supplies and required water quality measurements have been put into place.

*Cryptosporidium *is highly chlorine resistant and thus presents a challenge for its elimination from water supplies. Recent research on microbial resistance to treatment and disinfection demonstrates that the microbial surface structure and composition are key to determining the potential for waterborne transmission of emerging pathogens [25].

Commonly used methods for water disinfection in pipes rely on chlorination as the predominant method in part due to chlorine's wide range of activity and to its role as a lingering disinfectant, which unfortunately also leads to harmful residuals. Ultraviolet (UV) light has been shown in some cases to be equivalent to chlorine, but generates no disinfection residuals; the merit of such residuals is still in debate. Hammes and coworkers [26] showed that drinking water that had undergone ozonation and biological filtration, but which was distributed without any disinfectant residuals, had a high level of biological stability with total cell concentrations hardly changing throughout the distribution network. Their work highlights the benefit of utilizing multiple chemical and microbiological parameters to evaluate water quality since correlations could be found between organic carbon and cell counts, but not with HPC.

Treatment technologies need to increasingly address changes in water quality and quantity requirements along with secondary effects of usage of the treatments. Increased use of chlorine and chloramines has resulted in biofilm populations being supplemented with *Mycobacterium*. The increased usage of UV light has resulted in increased discharge of adenovirus in surface waters [27]; the super-surviving fraction has caused the disinfection microbe inactivation curves not to be linear [28]. Rotavirus has no significant inactivation with UV light from 100-350 mJ cm^2^. Changes in treatment technology will result in changes in the pathogens to which we are exposed, which will then lead to a problem of detection.

Straub and Chandler [29] addressed some of the fundamental challenges for detection of pathogens in water systems. Some of the key questions they raise include several that are still not fully answered: Is the detection objective enumeration, presence/absence, viability/infectivity; what is an appropriate level of risk (is it one viron per 1000 L of water); what volume of water needs to be or should be collected; is it necessary to detect the full range of likely pathogens or merely a few target organisms; and can the methods be automated.

Additionally, it will be necessary to take better account of historic weather patterns since the majority of all surface and ground water outbreaks are associated with heavy rainfall, much above normal levels [30]. As climate change leads to an increase in the frequency of storms and intensity of rainfall events, as well as an increase in the temperatures of surface and water distribution systems, an increase in the types and numbers of water-based pathogens will result. This will also likely cause an increase in taste and odor problems as a result of the expected increase in algal blooms.

To better prepare for changes in microbial water quality, treatment technologies with real time event monitoring where sensors are located throughout the distribution systems will be essential [31]. The primary challenge for such a system is that measurements must be performed in a continual, always on, manner and be capable of detecting low numbers of indicators and/or pathogens in large volumes of water (100-1000 L per minute). Commercial sensors are available for measurement of chemical water quality parameters including pH, chlorine, electrical conductivity, total organic or dissolved organic carbon, and turbidity. Few devices can detect microorganisms in a continual manner [32] and the ones that are available typically do not perform species identification (pathogenic vs. non-pathogenic strains).

In the past decade, PCR (polymerase chain reaction) based methods have become the most commonly used method for detection of waterborne viruses. PCR and RT-PCR methods have high specificity and sensitivity, can be used for nonculturable or noncytopathic viruses, and require a fairly short time for detection (generally 2-4 hours) [33]. Unfortunately, PCR methods can be limited for environmental analyses in that very small sample volumes are applied (which may lead to non-representative testing), the transcriptase enzymes can be inhibited by waterborne substances including humic acids, and the method cannot discriminate between infective and non-infective viruses. Persistence of infectivity can vary substantially across type of microbe and the environment [34]. Cell culture methods [35], when integrated with PCR may circumvent some of the problems [36,37] but require comparatively long times (4-30 days) for measurement.

Microchip based devices using antibodies can perform the required levels of detection, but their operation is typically very expensive, especially for a continual monitoring system, although some have promise for reusability [38]. Detection limits for some of these automated systems [39] are reported to be on the order of 10^4 ^cfu/mL; lower detection limits (more sensitive measurement) will be needed especially for measurement of viral contaminants having low requirements for infectivity. Biosensors have been developed that can perform capture of pathogens followed by real-time PCR using integrated waveguides [40]. Such optical methods have high promise for continual monitoring, however, without an enrichment or PCR step, detection limits tend to be higher than needed for municipal water monitoring. Nanoscale components using nanorods or nanoparticles can enhance signal strength and hence, reduce detection limits [41,42]; however stability and cost remain problematic. Antibody arrays [43] provide detection of multiple targets at one time; however, the broad-based microbial population of drinking water makes their implementation difficult.

Genomics, metagenomics, and functional genomics will provide information on exposure to pathogens to the level of being able to determine fairly precise locations of origin. Such source tracking has shown that there is more growing in the tap than one would think. Often such detailed analyses are not performed unless a contaminant made the water taste unacceptable or made someone ill. Such problems are likely to increase due to our over-reliance on an aging water infrastructure, from climate effects which increase surface water temperature, and due to unintended consequences of well-meaning applications of water treatment technologies.

For example, the water based pathogen *Naegleria fowleri *occurs in drinking water supply wells where the well temperature is between 20-40°C and feeds on HPC bacteria in the well. The use a more environmentally friendly biodegradable lubricant oil for well pump motors results in increased levels of HPC bacteria, increasing the numbers of *N. fowleri *[44]. *Naegleria *is one of the most resistant water-based pathogens to chlorine disinfectants and UV light; with 98% human mortality if infection occurs. Such a perfect microbial storm impacts how we have to consider unintended consequences of our desire to make a friendlier environment can affect our exposure to waterborne and water-based pathogens. Again, as we change the environmental conditions either through direct or indirect human action, the resultant microbial populations will find within their microenvironments new opportunities to thrive. We need to continuously develop new sources of viable drinking water.

Once pathogens have been detected in a water source, the water may be either discarded or the water itself and the conveyance systems must be treated to prevent recurrence. This is often accomplished by either removing the pathogens using a filtration step or by inactivating the pathogens using radiation or chemical, thermal, or other methods. Multiple-barrier or "hurdle" techniques are most efficient since no single technique can provide the necessary breadth of coverage.

Removal processes often involve passing the water through a coarse filtration system such as gravel or sand filters, which reduce gross turbidity. Such methods are especially effective for removing large diameter contaminants such as algal species and protozoa. Gravity-based settlement within a storage tank is often used but does require substantial holding times since pathogen-settling follows a terminal velocity based on particle diameter. A large, but typical protozoan measuring 250 um (Amoebic dysentery) will settle at a rate on the order of tens of mm per second. Such a simple process is often not sufficient by itself to meet the high pathogen removal level required.

Disinfection or inactivation methods provide a broader means to reduce pathogen load and typically are applied during a second stage. Heat and ultraviolet light treatments disrupt cellular proteins or genetic material in an irreversible manner but do not provide a lasting barrier against pathogen regrowth. Oxidative disinfection methods are effective at inactivating a broad range of pathogens. The effectiveness of such oxidative disinfection is species-specific and usually increases with the time and concentration applied. Chlorination or chloramination deliver a lasting effect but raise concerns for human health impact. Ozonation is highly effective but does not provide a lasting residual. Disinfection efficiency can vary substantially by the water chemistry (pH, salt, turbidity, etc.) and contact time. Unfortunately, disinfectants can react with naturally occurring materials in drinking water to form disinfection by-products (DBPs) which may lead to adverse health effects.

Filtration at the point of use (POU) is highly effective at removing pathogens and addresses concerns about water age. Water age refers to the time that an individual volume element exists in a water conveyance system and plays a major role in water quality deterioration within a distribution system [13]. As water travels through the distribution system it undergoes a variety of chemical and physical changes dependent on the flow rate, age and quality of the pipes and their deposited materials, finished water quality, and ambient temperature. Water age changes over the course of a day due in part to varying consumer demand changes. In general, a shorter water age correlates with few negative alterations in water quality. Water age has been determined to vary across cities based in part on the size and design of the conveyance system and the size of the population served with larger populations having shorter water ages. Regions with local populations over 500,000 generally have water ages on the order of 3-7 days while regions with smaller populations (less than 50,000) are estimated to have water ages of 12-24 days within the water conveyance system [13]. Water quality problems associated with water age include decay of disinfectants, formation of disinfection by-products, microbial regrowth (after decay of disinfectants), sediment deposition, and decreases in quality of taste and odor [13].

Removal of pathogens at the necessary high flow volumes is challenging. Commonly applied filtration suffers from membrane fouling thus motivating the search for new surfaces and coatings. One approach in development utilizes functionalized self-assembled monolayers [45] in which pathogens can be removed based on their electrostatic properties. In general, viruses will adsorb to negatively charged surfaces at pH values below the isoelectric point of the virus, which favors a positive surface charge [46]. If the water pH is above the pI (isoelectric point) of the microbe, then deposition is driven to oppositely charged surfaces. Reversal of the surface charge can in some cases provide a reversible means to remove the deposited microbe and regenerate the surface for future use [32]. Similarly, water pH and surface characteristics can impact transport of microbes through soils. This approach has been utilized to remove microbes from water by use of columns containing sand coated with ferric and aluminum hydroxides [47]. The amount of phosphate in the water (also a challenge for desalination) can influence bacterial adhesion by either reducing attachment (phosphate levels below 0.5 mM) or increasing attachment (phosphate levels above 0.5 mM) [48].

Many types of microorganisms have a natural tendency to attach to wet surfaces, where they then multiply and embed themselves in a slimy matrix forming a biofilm [49]. Biofilm formation on pipes and water conveyance systems contributes to the presence of HPCs in water systems and may play a role in hygiene problems. Since the attachment of microbes to surfaces and the development of biofilm phenotypes occur quickly, it is almost impossible to prevent biofilm formation [50]. The removal and killing of established biofilms require harsh treatments, mostly using oxidizing biocides which may have variable effectiveness depending on the nature of the biofilm. The emergence of bacteria resistant to conventional antimicrobials necessitates that new control strategies be developed. Some new methods employ the use of biological solutions (enzymes, phages, interspecies interactions, and antimicrobial molecules of microbial origin).

Generally, disinfectants do not penetrate the typical biofilm matrix remaining on a surface, such as a pipe, after an ineffective cleaning procedure [51]. Cleaning must be the first step applied and must be done to high levels of cleanliness; this is tremendously challenging for water distribution systems that are not designed for in-place cleaning, rinsing, or intermediate usage.

Enzyme-based detergents have been used as "green chemicals" to remove biofilms [49], especially in the food industry. Proteolytic enzymes have been successful, but are inhibited by the presence of other proteinaceous material. Introduction of surfactants improves the penetration into the biofilm.

Bacteriophages are viruses that infect bacteria and may provide a species-specific means to eliminate biofilms from pipes [52]. The biofilm bacteria would need to be susceptible to the phage. Most biofilms are comprised of a complex mixture of species and so application of this method has many challenges. A related approach utilizes introduction of microbial metabolites, which can interfere with the communal interactions and communications within a complex biofilm. This approach relies upon competition for substrates that can alter the balance of species within a biofilm [53].

## Desalination

There is a tremendous need for methods to substantially increase water supply, not only to meet current needs but to provide for population growth and increased water usage. Only 3% of water on our planet is fresh, but most of this is either in snow or ice or in aquifers that are challenging to reach. Extraction from groundwater often exceeds the rate of replenishment. An obvious solution for meeting global freshwater demand lies in the world's oceans, but this water is too salty to be used without extensive treatment such as that provided by desalination.

In Israel, where total demand for fresh water exceeds the average natural supply, the freshwater deficit is replaced to a great extent by treated wastewater used for agriculture [54,6]. However, the treatment of effluents prior to use in irrigation is not adequate for sustaining long-term agricultural production, due to salinization, which can deteriorate soil structure.

Sustainable production of adequate drinking water can only be achieved by desalination of sea and brackish waters [5,55]. Desalination is a process for extracting salt from seawater and returning water of a higher quality. Desalination is not a new idea and is already used in many regions, particularly in the Middle East. Saudi Arabia alone accounts for about a tenth of global desalination while Israel uses desalination technology to provide about a fourth of its domestic water needs. Such technologies must have lower impact on the environment, which means less energy use and reduced production of CO_2 _while utilizing the high salt effluent in ecologically sustainable ways.

Modern desalination plants employ reverse osmosis, which uses a membrane to separate out the salt. More than 12,000 desalination plants are now operational across the world. It has been estimated that up to 25 million m^3 ^of desalinated water is produced daily around the world [56]. Desalination facilities are expensive to build and require substantial amounts of energy to operate, making desalination suitable mainly for seaside cities in rich countries. It therefore has limited value for impoverished countries, where water supply problems are most serious.

Growing populations and political concerns are prompting governments and investors to look closely at desalination [55]. In a public sector partnership, Long Beach Water, along with the Los Angeles Department of Water & Power and the United States Bureau of Reclamation, has constructed a 300,000 gallon-per-day prototype desalination facility, the largest seawater desalination research and development facility of its kind in the United States. This facility was developed to evaluate new seawater desalination methods which could reduce cost and energy requirements.

Desalination by reverse osmosis has seen major improvements in the past 10 years. Further improvements, however, are likely to be incremental in part because of thermodynamic limitations [5,55]. A 10-20% reduction in energy use is potentially feasible [55]. Recoveries of seawater desalination are up to 50%, resulting in a brine discharge of environmental concern. Thermodynamics dictate a minimum energy of desalination, where the minimum energy needed to desalt water is independent of the technology or mechanism used for desalination [5].

New technologies that would decrease energy use, and therefore costs, might help desalination's contribution to drinking water supplies. Most of the research to reduce the energy use in reverse osmosis desalination has focused on creating high permeability (flux) membranes and low fouling membranes. The rationale for both improvements is to reduce the required hydraulic pressure and hence, the electric energy needed for the process. Membrane fouling is a sizeable problem, especially when the source water has high solids content or a substantial organic matter load. Most bacteria are removed by pre-treatment, but since the system is not sterile and because we cannot apply chlorine to RO membranes, biofilms will always grow. Such biofouling decreases the water flux, increases energy costs, and requires frequent membrane cleaning or replacement. *Bacillus *sp. and *Pseudomonas *sp. are among the most prevalent bacteria that form biofilms on RO and nanofiltration (NF) membranes used for water treatment [57]. Chlorination cannot be applied to reverse osmosis membranes because oxidants degrade the membrane structure [58] Inorganic scaling [54] can also arise due to phosphate and other inorganic ions present in wastewater effluent.

Nanotechnology has significant promise for use in reverse osmosis. Studies have shown that water flow through the inside of carbon nanotubes (so called nano-osmosis) yields flow rates that are 1,000 times to 10,000 times greater than expected based on the simple fluid mechanics calculations of flow in the pipe [5,59]. The surface inside such structures is smooth and hydrophobic and as a result, water flows as a string of molecules that readily slips through the membrane. Multi-carbon nanotubes have been used, but the diameter is too large for removing salt. Smaller single-walled carbon nanotubes (SWNTs) with a pore size of about 0.7 nm are critical for salt rejection, while a high density of nanotubes is needed for sufficient water productivity through such a membrane [60].

The nanotechnology approach has a biological inspiration for high flux membranes based on the aquaporin proteins extracted from living organisms, which can be incorporated into a lipid bilayer membrane or a synthetic polymer matrix [5]. While an intriguing approach, energy is needed even for membranes with infinite permeability since osmotic pressure of sea water, which is about 25 atm or 400 psi, must be overcome to induce permeation of fresh water through the reverse osmosis membranes. To get any meaningful water flux through the membrane one must exceed the pressure significantly. Additionally, the buildup of salt from the sea water next to the membrane leads to concentration polarization, requiring further energy to drive water through the membrane. The minimum theoretical energy cost for desalination of 50% recovery is 1 kWh/m^3 ^of water treated; however, considering practical limitations, it is likely not to be less than 1.5 kWh/m^3^. The long term goal is to achieve between 1.5-2 kWh/m^3 ^with a more likely energy cost of around 2 kWh/m^3^.

It is necessary to consider other approaches to desalinate salt water. In a process recently developed at Yale University, instead of applying hydraulic pressure, an osmotic pressure difference is used [61,62]. Ammonia and CO_2 _dissolved in water results in ammonium salts of very high concentration and hence, large osmotic pressure driving forces. This draw solution is used to induce natural osmotic flux of water across a semi-permeable membrane from a relatively dilute saline feedwater. The salts of this solution are thermolytic: when low-grade or waste heat is supplied, the salts decompose into ammonia and carbon dioxide gases for simple stripping from solution. These gases, when introduced to water again at a lower temperature, readily reconstitute the desired draw solutes. The major energy source for this "ammonia-carbon dioxide forward osmosis desalination process" is in the form of waste heat, with electric energy use of about 20% of that used in reverse osmosis desalination [63].

The environmental impact of desalination cannot be overlooked as there is a need to develop desalination technologies that consume less prime energy, typically electricity [55]. Methods for using waste heat (or recovering this heat) to desalinate sea water are needed to improve overall plant energy efficiency. Selection of the desalination facility is critical for minimizing ecological impact and can be hindered by contrasting goals. It is desirable to locate a facility far from any other discharge sources so that the intake will be minimally disrupted. Conversely, the plant discharge has lower ecological impact when it can be diluted with other waste streams having lower salinity [64].

The use and discharge of the concentrated brine must also be addressed in better ways so that the environmental impacts of larger and more numerous desalination plants are minimized. Desalination plant discharge can adversely affect aquatic communities due to substantial increases in salinity and the accumulation of metals, hydrocarbons and toxic anti-fouling compounds in receiving waters [65]. The ultimate consequence of this release can be shifts in the populations of plant and animal species due to the altered physical and chemical characteristics of the water [66]. Greater detail in monitoring is needed to ensure that ecological impacts lie within desired limits. However, many published monitoring studies lack sufficient detail with respect to study design and statistical analyses, making conclusive interpretation of results difficult [65].

Even with such advances it seems unlikely that desalination alone will be able to solve the world's water problems. Other approaches will be needed to reduce water use, to recycle what can be further used, and to capture new sources of water. These could include use of biological systems. Two such methods are discussed below.

Many halophyte plants and green algae have been used to desalinate water. Algae have been found in some of the harshest saline conditions on the planet and many are able to complete all of their life cycle processes in highly saline environments. Microalgae have been used in tertiary sewage treatment to eliminate nitrogen and phosphorous compounds [67]. Some microalgae, including *Scenedesmus obliquus *are able to use organic compounds and mixotrophic conditions [68]. Both fresh water and marine algal species can accumulate organic compounds as osmo-regulatory solutes in response to salt stress [69]. Salt-tolerant algal species are also being explored as producers of oil to generate biodiesel transportation fuels.

Microbial desalination of wastewater has been developed by the Bruce Logan Laboratory at Penn State University [70]. Typically desalination and wastewater treatment require substantial power input; however, Logan's approach replaces the power source with a microbial fuel cell. As the bacteria break down nutrients in the wastewater they also draw salt out of the water. Initial devices remove 88 to 94 percent of the salt, which is very good, but not yet as high as traditional desalination. Advances in electrodes and membrane materials along with stabilization of the microbial communities for use in a variety of environments will be necessary for full scale implementation.

## Other technologies to provide clean water

Technologies are being developed to improve recycling of wastewater and sewage treatment so that water can be applied for non-personal uses, such as irrigation, toilet flushing, or specific kinds of industrial purposes. Recycled water could resupply aquifers; however, very effective and consistent treatment methods along with rigorous safeguards are necessary to preserve the safety of recycled water. Advances in membrane technologies may lead to approaches, which can remove specific chemical and biological contaminants while passing along desired nutrients (N, P, and K in particular).

A different approach to the water problem involves developing strategies for reducing water use. Agricultural irrigation consumes enormous quantities of water; in developing countries, irrigation often exceeds 80 percent of total water use. Improved technologies that would more efficiently provide crops with water, such as sub-surface drip irrigation, (which is already commonplace in semi-arid regions of the U.S. and in the Middle East), can substantially reduce agricultural water demand. Despite its high efficiency, drip irrigation has been implemented only slowly in some areas due in part to the high cost of installation, to early problems (now solved) of fouling, and to general skepticism of a technology in which the critical input water is not seen by the farmer.

Methodologies are needed for small scale purification of water, especially in the developing world. Wide-spread use of inexpensive gravity-fed filtration units, solar distillation, and low-power treatment systems could greatly reduce waterborne disease [6,7]. The ultimate cost to the consumer and ease of use and maintenance, especially in low-income rural areas, are key factors at least as important as technological design requirements. Cheap and efficient techniques using "homemade" filter media, such as slow sand filtration in rural areas, are being tested for their ability to remove chemical and biological contaminants [71]. It is necessary that methods not only be effective, but that they be cost effective and maintained by personnel with only minimal training [71].

## Summary

To meet the NAE's grand challenge of providing safe drinking water requires advances in technology to provide safe and substantial quantities of water in addition to addressing societal problems tied to socioeconomics. Removal or inactivation of pathogens are key steps to prevent widespread disease. However, outbreaks of waterborne and water-based pathogens remain problematic even in the developed world. As reserves of accessible fresh water continue to be depleted, desalination techniques will become increasingly important. While thermodynamic limits have nearly been met using advanced nanotechnologies, further refinement to reduce biofouling can decrease total energy costs while increasing sustainability.

We need new indicator microorganisms that are water based pathogens and real time sensors that are capable of organism identification. Detection sensitivity is needed to be able to find 1 organism/1-10,000 mL, along with some assessment of viability. These are great challenges, but we also need technologies for treatment of biofilms in the distribution system. We need technologies for treatment at the tap and for premise plumbing, as well as new disinfectants, fewer by-products, and disinfectants designed to work in conjunction with existing disinfectants.

To this end, the major questions addressed include the following:

1) How do multi-scale, natural and engineered systems interact and subsequently affect water quality and quantity?

2) How can the fate of chemical and biological water contaminants be monitored, modeled, and controlled? Similarly, should emphasis be placed on detection and monitoring methods of high specificity or broad applicability?

3) How can the energy and CO_2 _burden of purification methods, including desalination, be provided for in a sustainable manner?

4) How can socioeconomic burdens related to access to safe drinking water in the developing world be reduced?

Fully addressing this grand challenge will continue to be a task for engineers and scientists developing and applying a multitude of methods. These challenges are met by advances made at the interface of what are commonly considered scientific and engineering disciplines and can benefit greatly by fully integrating biological components and accounting for biological impacts of engineering design decisions.

## Competing interests

The authors declare that they have no competing interests.

## Authors' contributions

MR developed the structure of the manuscript and much of the text; CG provided information primarily on microbial technologies; ME provided information primarily on desalination. All authors read and approved the final manuscript.
